# Non-Linear Association Between Serum Alkaline Phosphatase and 3-Month Outcomes in Patients With Acute Stroke: Results From the Xi'an Stroke Registry Study of China

**DOI:** 10.3389/fneur.2022.859258

**Published:** 2022-07-15

**Authors:** Weiyan Guo, Zhongzhong Liu, Qingli Lu, Pei Liu, Xuemei Lin, Jing Wang, Yuanji Wang, Qiaoqiao Chang, Fang Wang, Songdi Wu

**Affiliations:** ^1^Department of Neurology, The First Affiliated Hospital of Northwest University, Xi'an No.1 Hospital, Xi'an, China; ^2^Department of Epidemiology and Biostatistics, School of Public Health of Xi'an Jiaotong University Health Science Center, Xi'an, China

**Keywords:** acute stroke, alkaline phosphatase, outcomes, risk factor, China

## Abstract

**Background:**

Alkaline phosphatase (ALP) is associated with an increased risk of cardiovascular events and is closely related to adverse outcomes after stroke. However, the regional investigation into the associations of ALP with acute stroke (AS) outcomes is limited. This study aimed to identify the association between serum ALP levels and clinical outcomes 3 months after AS in the Xi'an district of China.

**Methods:**

We enrolled all patients with AS from 4 hospitals in the Xi'an district from January to December 2015. ALP levels and related patient information were collected at admission, and the events of stroke outcomes were followed up 1 and 3 months after diagnosis. ALP levels were analyzed as continuous variables and quartiles (Q1–Q4). The outcomes included all-cause mortality, recurrent stroke, and poor functional outcomes (modified Rankin Scale score of 3–6) within 3 months. A multivariate logistic regression and interaction analyses were performed to evaluate the independent association between serum ALP level and 3-month stroke outcomes.

**Results:**

Overall, 2,799 patients with AS were enrolled in this study. The mean age was 63.9 ± 12.5 years. In the Q4 (≥93.0 U/L) group, the incidences of all-cause mortality, recurrent stroke, and poor functional outcomes were 7.8, 2.7, and 24.9%, respectively. After being adjusted for confounding variables, patients in Q4 (≥93.0 U/L) were related to an increased risk of all-cause mortality [odds ratio (OR) = 2.17, 95% CI: 1.19–3.96; *P* = 0.011] and patients in Q3 (76.8–92.9 U/L) were related to a lower risk of recurrent stroke (OR = 0.37, 95% CI: 0.14–0.97; *P* = 0.043) at the 3-month time point, compared to those in Q2 (63.0–76.7 U/L). The optimal range of ALP for all-cause mortality was seen in Q2, with a nadir level of 70 U/L. However, differences were statistically insignificant between ALP levels and poor functional outcomes (*P* > 0.05). Moreover, there was no significant interaction between ALP levels and age, gender, drinking status, smoking status, or pneumonia (*P* > 0.05) for all outcomes.

**Conclusion:**

Non-linear associations were observed between serum ALP levels and 3-month outcomes in patients with AS. It might be beneficial to reduce the risk of all-cause mortality and recurrent stroke by maintaining ALP at optimal ranges.

## Introduction

Strokes are a leading cause of death and disability, ranking third globally and first in China (1, 2). Therefore, there is an urgent need to identify and manage the risk factors for adverse stroke outcomes as early as possible.

Alkaline phosphatase (ALP) is an enzyme that catalyzes the hydrolysis of organic pyrophosphate, an inhibitor of vascular calcification (3, 4). Serum ALP is a well-known marker of skeletal or hepatobiliary dysfunction in clinical practice (5–7), the functions of ALP include regulation of the balance between initiators and inhibitors of mineralization, and enhancement of vascular calcification (8, 9). In recent years, there has been increasing evidence that serum ALP level is related to the risk of cardiovascular diseases and all-cause mortality in the general population, myocardial infarction, survivors and clinical populations (10–17). Previously, some other studies revealed that ALP was associated with stroke outcomes. Two investigations reported that elevated ALP was related to all-cause mortality or stroke recurrence after stroke (18, 19), and another two studies revealed positive relationships between ALP and poor functional outcomes after ischemic and hemorrhagic stroke (20, 21). However, the above investigations were mainly large-scale or single-center studies, and there is a lack of small-scale, regional, or multi-center studies (18–21).

In this study, we collected serum ALP and related clinical data of patients with acute stroke (AS) in the Xi'an Stroke Registry Study of China, hoping to investigate the associations between serum ALP levels and 3-month clinical outcomes including all-cause mortality, recurrent stroke, and poor functional outcomes in patients with AS.

## Materials and Methods

### Study Population

**Figure 1:**
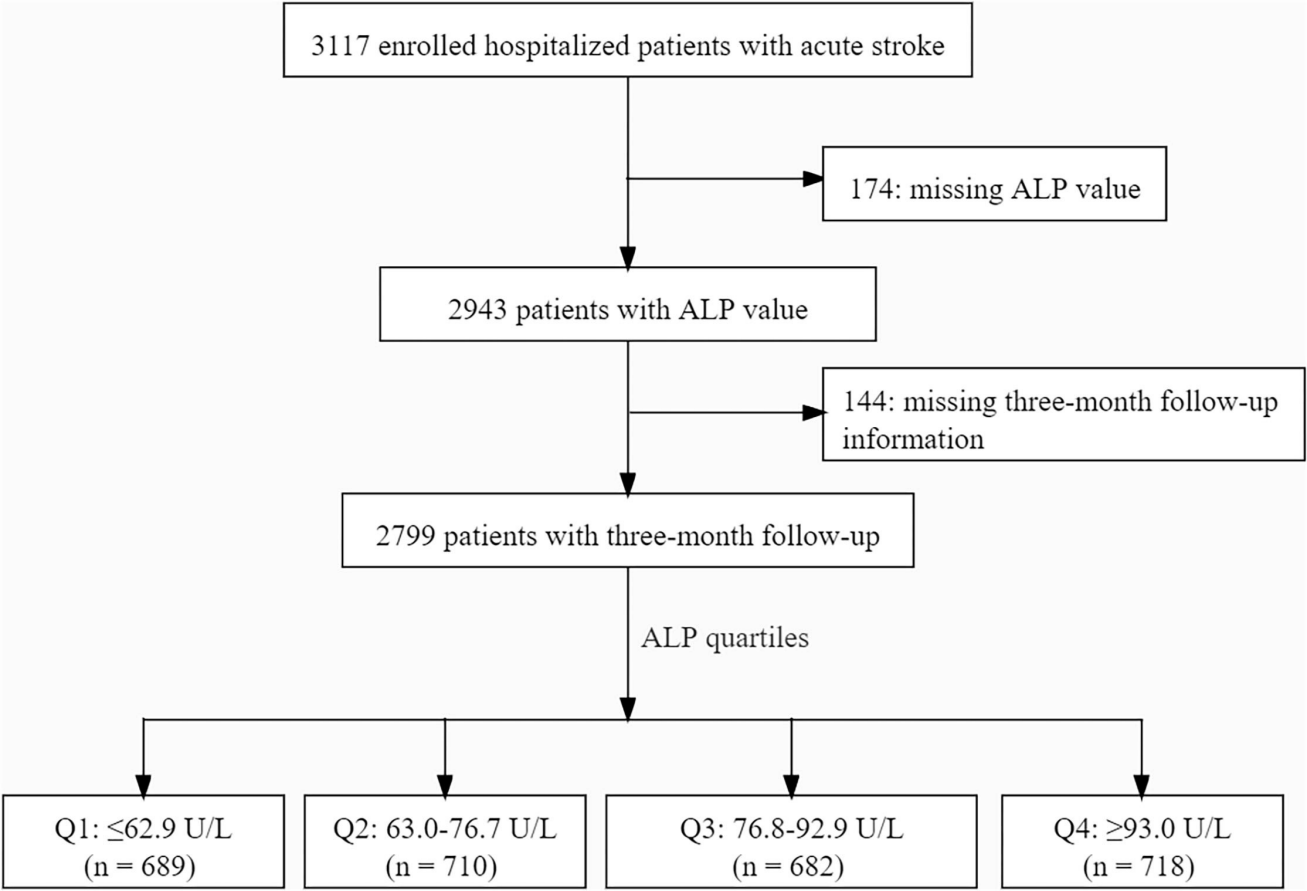
Flowchart of patient enrollment. ALP, alkaline phosphatase.

All patients with AS hospitalized in any of the four tertiary grade A hospitals in the Xi'an district of China between January and December of 2015 were enrolled. Patients were eligible if they met the following criteria: (1) 18 years of age or older; (2) diagnosed within 7 days after symptom onset; (3) diagnosed as acute ischemic stroke, transient ischemic attack, spontaneous intracerebral hemorrhage, or subarachnoid hemorrhage confirmed by computed tomography or magnetic resonance imaging. During the initial stage of the study, 3,117 patients with AS underwent integrated medical examinations, and follow-ups were conducted one and 3 months after stroke onset. Among these patients, 174 without ALP values were excluded. Furthermore, 144 patients lost to follow-up at the end of 3 months were also excluded. The resulting cohort included 2,799 patients with AS for the final analysis. The details of the inclusion and exclusion criteria and the study process flowchart are illustrated in Figure 1. The inclusion criteria were consistent across all participating hospitals. The study was conducted following the guiding principles of the Declaration of Helsinki. The academic committee of Xi'an No. 1 Hospital and the ethics committees of all participating hospitals approved the study [Approval No. 2014(5)]. Written informed consent was obtained from all patients.

### Baseline Data Collection

This is a multi-center, prospective cohort study to investigate the association between serum ALP levels and outcomes of AS patients in Xi'an, China. Baseline data, including demographic information, medical history, vascular risk factors, assessment at admission and discharge, important laboratory data, and complications, were collected (Table 1). Vascular risk factors included previous stroke, hypertension, diabetes mellitus (DM), atrial fibrillation (AF), smoking status, and drinking status. Body mass index (BMI) and other related complications were defined according to the Chinese National Stroke Registry (CNSR) study (22). The National Institutes of Health Stroke Scale (NIHSS) was used to estimate the initial neurologic severity within 24 h of admission. The occurrence of pneumonia during hospitalization was also recorded. Fasting blood samples were drawn within 24 h of admission, and routine laboratory tests were conducted within 2 h of collection. Using an automated enzymatic method, serum ALP levels were measured at each research center. All measurements were performed in a central laboratory blinded to the clinical situations of all subjects. The ALP levels were analyzed and treated as continuous variables and categorical variables (Q1–Q4). ALP quartiles (Q1–Q4) were defined as the distribution of ALP from low to high and divided into four parts. The range of quartiles (Q1–Q4) are as follows: Q1: ≤ 62.9 U/L, Q2: 63.0–76.7 U/L, Q3: 76.8–92.9 U/L, Q4: ≥ 93.0 U/L.

**Table 1:** Baseline characteristics of the patients according to quartiles of ALP levels.

**Characteristics**	**Overall** **(*n* = 2,799)**	**ALP quartile, U/L**				***P*-value**
		**Q1 (≤62.9);** **(*n* = 689)**	**Q2 (63.0–76.7);** **(*n* = 710)**	**Q3 (76.8–92.9);** **(*n* = 682)**	**Q4 (≥93.0);** **(*n* = 718)**	
Age (mean ± SD), y	63.9 ± 12.5	64.3 ± 13.1	63.9 ± 12.2	63.9 ± 12.1	63.5 ± 12.4	0.719
Female	1,058 (37.8)	230 (33.4)	253 (35.6)	260 (38.1)	315 (43.9)	<0.001
**Vascular factors**						
Smoking, *n* (%)						0.002
Never smoking	1,589 (56.8)	392 (56.9)	398 (56.1)	378 (55.4)	421 (58.6)	
Smoking cessation	539 (19.3)	155 (22.5)	137 (19.3)	144 (21.1)	103 (14.3)	
Current smoking	671 (24.0)	142 (20.6)	175 (24.6)	160 (23.5)	194 (27.0)	
Moderate to heavy alcohol consumption, *n* (%)	664 (23.7)	167 (24.2)	195 (27.5)	162 (23.8)	140 (19.5)	0.005
Previous stroke, *n* (%)	769 (27.5)	207 (30)	195 (27.5)	185 (27.1)	182 (25.3)	0.267
Hypertension, *n* (%)	2,004 (71.6)	467 (67.8)	503 (70.8)	511 (74.9)	523 (72.8)	0.024
DM, *n* (%)	603 (21.5)	137 (19.9)	154 (21.7)	153 (22.4)	159 (22.1)	0.658
AF, *n* (%)	185 (6.6)	48 (7.0)	41 (5.8)	46 (6.7)	50 (7.0)	0.775
NIHSS score at admission, median (IQR)	4.0 (2.0, 7.0)	4.0 (2.0, 6.0)	4.0 (1.0, 6.0)	4.0 (2.0, 6.0)	4.0 (2.0, 9.0)	<0.001
Pneumonia during hospitalization, *n* (%)	188 (6.7)	48 (7.0)	30 (4.2)	49 (7.2)	61 (8.5)	0.012
**Laboratory findings**						
TC, mmol/L, mean ± SD	4.4 ± 1.0	4.2 ± 1.1	4.4 ± 1.1	4.3 ± 1.0	4.5 ± 1.1	<0.001
TG, mmol/L, mean ± SD	1.7 ± 1.3	1.6 ± 1.5	1.6 ± 1.3	1.7 ± 1.3	1.7 ± 1.1	0.419
HDL-C, mmol/L, mean ± SD	1.1 ± 0.3	1.1 ± 0.3	1.1 ± 0.3	1.1 ± 0.3	1.2 ± 0.3	0.119
LDL-C, mmol/L, mean ± SD	2.6 ± 0.8	2.5 ± 0.8	2.6 ± 0.8	2.6 ± 0.8	2.7 ± 0.9	<0.001
FBG, mmol/L, mean ± SD	6.0 ± 2.4	5.7 ± 2.1	6.0 ± 2.4	6.1 ± 2.4	6.3 ± 2.7	<0.001
ALT, U/L, median (IQR)	19.0 (14.0–28.0)	17.4 (13.0–25.0)	19.0 (14.0–27.0)	19.0 (14.0–29.0)	21.0 (15.0–30.0)	<0.001
AST, U/L, median (IQR)	21.3 (17.0–28.0)	20.3 (16.2–27.0)	21.0 (17.0–26.0)	22.0 (17.0–29.0)	23.0 (18.0–31.0)	<0.001
Cr, mg/L, mean ± SD	0.8 ± 0.4	0.8 ± 0.3	0.8 ± 0.3	0.8 ± 0.3	0.9 ± 0.7	0.107
eGFR, mean ± SD	75.1 ± 17.8	75.9 ± 17.0	75.0 ± 18.4	75.1 ± 17.9	74.4 ± 17.9	0.508
BUN, mmol/L, mean ± SD	5.2 ± 1.9	5.1 ± 1.9	5.0 ± 1.8	5.1 ± 1.9	5.3 ± 2.1	0.025
INR, mean ± SD	1.0 ± 0.2	1.0 ± 0.2	1.0 ± 0.3	1.0 ± 0.1	1.1 ± 0.2	0.121
UA, μmol/L, mean ± SD	284.0 ± 99.7	285.1 ± 102.9	287.4 ± 98.3	286.4 ± 95.9	277.5 ± 101.4	0.233
WBC counts, ×10^12^/L, mean ± SD	7.2 ± 2.7	6.9 ± 2.6	7.1 ± 2.5	7.2 ± 2.6	7.6 ± 3.1	<0.001
PLT counts, ×10^9^/L mean ± SD	188.1 ± 60.8	183.7 ± 57.6	192.4 ± 59.2	189.2 ± 62.6	187.0 ± 63.4	0.057
BMI on admission, kg/m^2^, mean ± SD	23.8 ± 3.5	24.0 ± 4.0	24.0 ± 3.4	23.8 ± 3.3	23.6 ± 3.1	0.142
SBP on admission, mmHg, mean ± SD	147.4 ± 23.0	145.0 ± 21.7	146.6 ± 21.8	148.8 ± 23.6	149.1 ± 24.7	0.002
DBP on admission, mmHg, mean ± SD	86.6 ± 13.3	85.3 ± 12.5	85.7 ± 12.8	87.3 ± 13.5	88.3 ± 14.3	<0.001
Pulse, bmp, mean ± SD	75.1 ± 11.2	73.9 ± 10.6	74.8 ± 10.6	75.5 ± 11.2	76.1 ± 12.4	0.002
NHISS score at discharge, median (IQR)	2.0 (0.0, 4.0)	2.0 (0.0, 4.0)	2.0 (0.0, 4.0)	2.0 (1.0, 4.0)	2.5 (0.0, 5.0)	0.017
mRS score at discharge, *n* (%)						<0.001
mRS score ≤ 2	2,147 (76.7)	555 (80.5)	568 (80.0)	512 (75.1)	512 (71.3)	
mRS score 3–6	652 (23.3)	134 (19.5)	142 (20.0)	170 (24.9)	206 (28.7)	
Stroke types						0.042
Ischemic stroke	2,238 (80.0)	563 (81.7)	580 (81.7)	546 (80.1)	549 (76.5)	
Transient ischemic attack	194 (6.9)	53 (7.7)	52 (7.3)	42 (6.2)	47 (6.5)	
Spontaneous intracerebral hemorrhage	245 (12.3)	69 (10)	73 (10.3)	88 (12.9)	115 (16.0)	
Subarachnoid hemorrhage	22 (0.8)	4 (0.6)	5 (0.7)	6 (0.9)	7 (1.0)	

*ALP, alkaline phosphatase; Q, quartile; DM, diabetes mellitus; AF, atrial fibrillation; NIHSS, National Institutes of Health Stroke Scale; IQR, interquartile range; TC, total cholesterol; TG, triglyceride; HLD-C, high-density lipoprotein cholesterol; LDL-C, low-density lipoprotein cholesterol; FBG, fasting blood glucose; ALT, alanine aminotransferase; AST, aspartate aminotransferase; Cr, creatinine clearance rate; eGFR, estimated glomerular filtration rate; BUN, blood urea nitrogen; INR, international normalized ratio; UA, uric acid; WBC, white blood cell; PLT, platelet; BMI, body mass index; SBP, systolic blood pressure; DBP, diastolic blood pressure; mRS, modified Rankin Scale*.

### Clinical Outcomes and Follow-Up

A follow-up was performed 1 and 3 months after the onset of AS. Patients were followed up over the phone or interviewed face-to-face by trained research coordinators, who were unaware of the stroke history of all patients. The adverse clinical outcomes of the 3-month follow-up included all-cause mortality, recurrent stroke, and poor functional outcomes. All-cause mortality was defined as death from any cause and confirmed through the medical records from the hospitals where the treatments or the death certificates of the local citizen registry were issued. Recurrent stroke included the new occurrence of ischemic stroke, transient ischemic attack, spontaneous intracranial hemorrhage, or subarachnoid hemorrhage during the follow-up. Confirmation of recurrent stroke was sourced from the corresponding hospitals to ensure a reliable diagnosis during the follow-up. In the case of suspected recurrent stroke events without hospitalization, adjudication was made by the independent outcome events judgment committee. A modified Rankin Scale score of 3–6 3 months after AS onset was defined as a poor functional outcome.

### Statistical Analysis

Continuous variables were presented as mean ± SD or medians (interquartile range, IQR) and categorical variables were presented as percentages (%). One-way analysis of variance (ANOVA) was used for normally distributed continuous variables to assess differences between groups, and a chi-square test (χ^2^) was used for categorical variables. When the sample did not satisfy a normal distribution, multiple groups were compared using the Kruskal–Wallis rank-sum test. Multivariable logistic regression models were performed to investigate the correlation between serum ALP levels and stroke outcomes. Odds ratios (ORs) and 95% CIs were calculated. The covariables in the logistic regression equation were selected based on their associations with the outcomes of interest or a change in effect estimate of >10%. The potential association pattern between ALP and 3-month stroke outcomes was explored on a continuous scale with restricted cubic splines. A two-piecewise linear regression model was performed to examine the threshold effect of ALP on stroke outcomes in terms of the smoothing plot. A recurrence method was used to automatically calculate the breaking point of ALP, yielding the maximum model likelihood. A log-likelihood ratio test was also conducted to compare the one-line linear model with the two-piecewise linear regression model. Kaplan–Meier curves (log-rank test) were used to evaluate the difference in the probability of 3-month outcomes among different stroke types. Subgroup analyses were conducted for age, gender, smoking status, drinking status, and pneumonia during hospitalization using stratified logistic regression models. Interactions among subgroups were tested using a likelihood ratio test. Statistical significance was set at *P* <0.05. All analyses were conducted using the statistical software packages R 3.3.2 (http://www.R-project.org, The R-Foundation) and Free Statistics software version 1.3 (Free Clinical Medical Technology, Inc. Beijing, China).

## Results

### Baseline Characteristics

A total of 2,799 eligible subjects (1,741 men and 1,058 women) were enrolled in our study, with a mean age of 63.9 ± 12.5 years. In the higher quartiles of ALP, there were more women, more patients with hypertension, smokers, and nondrinkers, and more patients with pneumonia during hospitalization. Higher baseline NHISS scores existed. Patients from the higher quartiles tended to have higher triglyceride levels, low-density lipoprotein, fasting blood glucose, alanine aminotransferase, aspartate aminotransferase, blood urea nitrogen, white blood cell count, systolic blood pressure, diastolic blood pressure, and pulse. However, the levels of serum total cholesterol, high-density lipoprotein, creatinine clearance rate, estimated glomerular filtration rate, international normalized ratio, uric acid, and platelet did not increase with serum ALP levels. Age, previous stroke, DM, AF, and BMI were not significantly different among the quartiles. The distributions of the different stroke types are illustrated in Table 1.

### The Rates of 3-Month Outcomes Grouped by Quartiles of ALP

The clinical outcomes at the 3-month time point are illustrated in Table 2. The overall incidences of all-cause mortality, recurrent stroke, and poor functional outcomes at 3 months were 4.6, 2.0, and 20.4%, respectively. The rates of all-cause mortality and poor functional outcome at 3 months were higher in the Q4 group when compared with the Q1, Q2, and Q3 groups (*P* < 0.001). However, there was no significant difference in recurrent stroke among different ALP quartiles (*P* = 0.097).

**Table 2:** Rates of 3-month outcomes according to quartiles of ALP levels.

**Outcomes**	**Overall** **(*n* = 2,799)**	**ALP quartile, U/L**				***P*-value**
		**Q1(≤62.9)**	**Q2 (63.0–76.7)**	**Q3 (76.8–92.9)**	**Q4 (≥93.0)**	
All-cause mortality, *n* (%)	130 (4.6)	25 (3.6)	21 (3)	28 (4.1)	56 (7.8)	<0.001
Recurrent stroke, *n* (%)	55 (2.0)	14 (2.1)	16 (2.3)	6 (0.9)	19 (2.7)	0.097
Poor functional outcome, *n* (%)	571 (20.4)	119 (17.3)	121 (17)	152 (22.3)	179 (24.9)	<0.001

*ALP, alkaline phosphatase; Q, quartile*.

### Multivariable Logistic Regression of 3-Month Outcomes

The multivariable logistic regression analysis of ALP levels and stroke outcomes are illustrated in Table 3. A continuous variable analysis demonstrated that the risk of death increases by 7% after being adjusted for potential confounding variables (adjusted OR = 1.07, 95% CI: 1.01–1.14; *P* = 0.03) when ALP rises per 10 U/L. However, the risk of recurrent stroke (adjusted OR = 1.00, 95% CI: 0.91–1.10; *P* = 0.978) and poor functional outcomes (adjusted OR = 1.04, 95% CI: 0.98–1.08; *P* = 0.086) did not increase with ALP levels.

**Table 3:** Multivariable logistic regression of 3-month outcomes.

**Variable**	**Overall, *n***	**Event, *n* (%)**	**Crude** **OR (95%CI)**	***P*-value**	**Adjusted^**a**^** **OR (95% CI)**	***P*-value**
**All-cause mortality**						
ALP, per 10-unit increase	2,799	130 (4.6)	1.13 (1.07–1.18)	<0.001	1.07 (1.01–1.14)	0.03
ALP quartile						
Q1	689	25 (3.6)	1.24 (0.68–2.23)	0.483	1.42 (0.72–2.78)	0.31
Q2	710	21 (3)	Reference		Reference	
Q3	682	28 (4.1)	1.4 (0.79–2.50)	0.247	1.37 (0.71–2.63)	0.351
Q4	718	56 (7.8)	2.78 (1.66–4.63)	<0.001	2.17 (1.19–3.96)	0.011
*P* for trend				<0.001		0.051
**Recurrent stroke**						
ALP, per 10-unit increase	2,799	55 (2)	1.03 (0.94–1.13)	0.542	1.00 (0.91–1.10)	0.978
ALP quartile						
Q1	689	14 (2)	0.89 (0.43–1.83)	0.745	0.95 (0.45–2.03)	0.903
Q2	710	16 (2.3)	Reference		Reference	
Q3	682	6 (0.9)	0.38 (0.15–0.98)	0.046	0.37 (0.14–0.97)	0.043
Q4	718	19 (2.6)	1.18 (0.60–2.31)	0.632	0.99 (0.48–2.04)	0.976
*P* for trend				0.793		0.684
**Poor functional outcome**						
ALP, per 10-unit increase	2,799	571 (20.4)	1.08 (1.04–1.11)	<0.001	1.04 (0.98–1.08)	0.086
ALP quartile						
Q1	689	119 (17.3)	1.02 (0.77~1.34)	0.91	0.96(0.69–1.34)	0.811
Q2	710	121 (17)	Reference		Reference	
Q3	682	152 (22.3)	1.4 (1.07–1.82)	0.014	1.22 (0.89–1.69)	0.221
Q4	718	179 (24.9)	1.62 (1.25–2.09)	<0.001	1.18 (0.85–1.63)	0.316
*P* for trend				<0.001		0.122

^a^*Adjusted for age, gender, smoking, drinking, previous stroke, National Institutes of Health Stroke Scale score, pneumonia during hospitalization, estimated glomerular filtration rate, white blood cell, body mass index, hypertension, diabetes mellitus, and atrial fibrillation*.

*ALP, alkaline phosphatase; Q, quartile; OR, odds ratio; CI confidence interval*.

A quartile variable analysis indicated that patients in Q4 were more likely to have a higher risk of mortality within 3 months of AS (adjusted OR = 2.17, 95% CI: 1.19–3.96, *P* = 0.011) when compared with those in Q2. Patients in Q1 and Q3 were related to a higher risk of all-cause mortality than those in Q2, but there was no significant difference was revealed (*P* > 0.05). For recurrent stroke, patients in Q3 were correlated with a lower risk of recurrent stroke compared to those in Q2 (adjusted OR = 0.37, 95% CI: 0.14–0.97; *P* = 0.043). Meanwhile, patients in Q1 and Q4 tended to have a relatively lower risk of recurrent stroke, but the difference was insignificant (*P* > 0.05) in comparison with those in Q2. In comparison with Q2, the risk of poor functional outcomes was lower in Q1 and higher in Q3 and Q4, but these differences were not significant (*P* > 0.05).

We further conducted a threshold effect analysis of ALP level with 3-month all-cause mortality and recurrent stroke. The two-piecewise linear regression model demonstrated that the optimal range for all-cause mortality was Q2, with a nadir value of 70 U/L (Log-likelihood ratio test, *P*-value 0.035; Table 4), and the optimal range for recurrent stroke was Q3, with a nadir value of 80 U/L (Log-likelihood ratio test, *P*-value 0.119; Supplementary Table I).

**Table 4:** Threshold effect analysis of ALP level and 3-month all-cause mortality.

**Outcomes**	**OR (95% CI)**	***P*-value**
One-line linear regression model (per 10-unit increase)	1.07 (1.01–1.14)	0.030
Two piecewise linear regression model		
ALP <70 U/L	0.97 (0.95–1.01)	0.143
ALP > 70 U/L	1.02 (1.01–1.03)	0.017
Log-likelihood ratio test		0.035

*Adjusted for age, gender, smoking, drinking, previous stroke, National Institutes of Health Stroke Scale score, pneumonia during hospitalization, estimated glomerular filtration rate, white blood cell, body mass index, hypertension, diabetes mellitus and atrial fibrillation*.

*ALP, alkaline phosphatase; OR, odds ratio; CI, confidence interval*.

**Figure 2:**
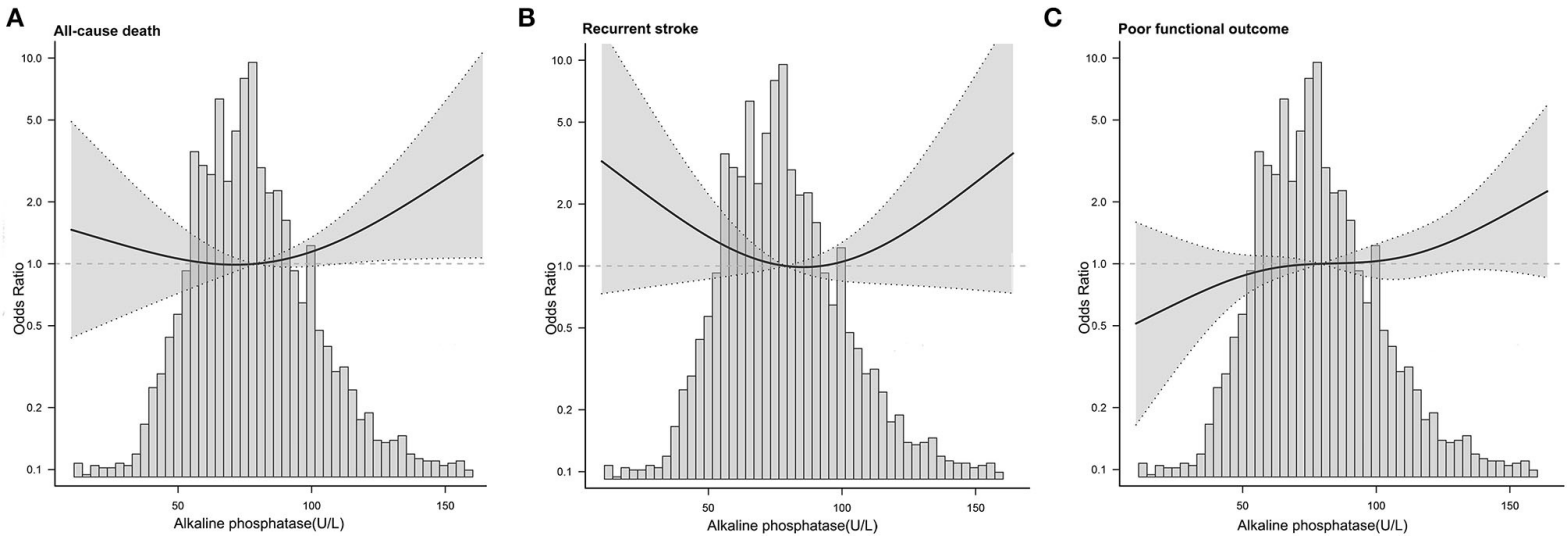
Adjusted odds ratio of adverse outcome after acute stroke according to serum ALP levels on admission. **(A)** All-cause mortality. **(B)** Recurrent stroke. **(C)** Poor functional outcome.

In addition, the functional outcomes of different stroke types were analyzed, and all four stroke subtypes had lower proportions of poor functional outcomes (Supplementary Figure I). We further conducted a logistic regression analysis of the association between ALP and poor functional outcomes in different stroke subtypes and the results showed no significant differences after adjusting for confounding variables (*P* > 0.05, Supplementary Table II). And the association was stable in different stroke subtypes (*P* for interaction > 0.05, Supplementary Table II). In addition, non-linear associations were all revealed between serum ALP levels and 3-month outcomes after AS by restricted cubic splines (Figure 2).

### Subgroup Analysis

To assess whether the associations between ALP levels and 3-month outcomes among the different subgroups were consistent, stratified and interactive analyses were performed. The analyses demonstrated that age, gender, smoking status, drinking status, and pneumonia during hospitalization had no interaction effect on the associations between ALP levels and adverse stroke outcomes, including all-cause mortality, recurrent stroke, and poor functional outcomes (*P* for interaction ≥0.05, Supplementary Figure II).

In addition, to preliminarily identify the cumulative rates of all-cause mortality and recurrent stroke in different stroke subtypes, we plotted the Kaplan–Meier curves of 3-month stroke outcomes for different subtypes. Kaplan–Meier curves demonstrated that spontaneous intracerebral hemorrhage and subarachnoid hemorrhage might have higher mortality rates than TIA and ischemic stroke, while only subarachnoid hemorrhage had the highest rate of recurrent stroke within 3 months (*P* < 0.001; Supplementary Figure III).

## Discussion

This study found non-linear associations between serum ALP levels and outcomes at the 3-month time point in patients with AS in the Xi'an district. The optimal range for all-cause mortality was Q2 (63.0–76.7 U/L) with a nadir value of 70 U/L. As for recurrent stroke, the preferable range was Q3 (76.8–92.9 U/L).

Previously, a CNSR-based study demonstrated that the higher ALP levels (>98 U/L) had a 1.36-fold increased risk of 1-year all-cause mortality when compared with the lower ALP levels (≤ 59.0 U/L) (18). A prospective study from South Korea revealed that the higher ALP levels (≥97 U/L) were related to a 2.83-fold increased risk of long-term all-cause mortality in contrast to the lower ALP levels (≤ 57.0 U/L) (19). In our results, the risk of all-cause mortality at 3 months for the Q4 group (≥93.0 U/L) increased 2.17 times compared with the Q2 group (63.0–76.7 U/L). The risk of all-cause mortality obtained in this study was higher than that in the CNSR-based study but lower than that in the South Korean study. This discrepancy might be due to differences in regions, study populations, and follow-up periods. In contrast to our study, the CNSR-based study ruled out patients with eGFR <60 ml/min per 1.73 m^2^ and had a 1-year follow-up. The eGFR was adjusted as a covariate to control the influence of renal impairment in our study but was not adjusted in the South Korean study. Additionally, the optimal ALP value and breakpoint were limited, as referenced in previous studies (18, 19). The threshold effect and two piecewise linear regression analysis of our study indicate that both lower and higher levels of ALP were associated with an elevated risk of all-cause mortality and that the optimal range was Q2: 63.0–76.7 U/L with a nadir value of 70 U/L. These results suggest that the risk of all-cause mortality in stroke patients in the Xi'an district may be reduced by maintaining optimal ALP levels.

Elevated ALP levels were also considered to be related to an increased incidence of recurrent stroke. The CNSR-based study also suggested that a higher ALP level (>98 U/L) was associated with a 1.45-fold higher risk of 1-year recurrent stroke when compared with the lower ALP levels (≤ 59.0 U/L) (18). Our study demonstrated that the risk of recurrent stroke decreased by 63% in Q3 (76.8–92.9 U/L), compared with the ALP levels in Q2 (63.0–76.7 U/L). In contrast to the CNSR-based study, our study revealed a strong non-linear association between ALP levels and 3-month recurrent stroke with an optimal range in Q3 (76.8–92.9 U/L). Our results were similar to those of a Japanese study, in which a non-linear association between ALP and stroke occurrence was found (12). In addition to differences in follow-up periods, populations, and research designs, we considered that the differences in the baseline characteristics of patients might also account for the discrepancy with the CNSR-based study. In Xi'an district, fewer patients with previous stroke (27.5 vs. 34.0%), smokers (24.0 vs. 43.2%) and drinkers (23.7 vs. 28.5%) were detected. The unique characteristic of patients in the Xi'an district highlights the importance of conducting regional stroke research. These differences also indicate that the characteristics of stroke patients may differ by regions, lifestyles, and economic statuses, emphasizing the importance of conducting regional stroke research (2, 23). The risk of short-term recurrent stroke might be reduced by monitoring and maintaining serum ALP levels at a preferable range at early onset.

Previous studies have demonstrated that higher serum ALP levels are related to poor functional outcomes (8, 18, 20). However, our data revealed an insignificant association between ALP levels and 3-month poor functional outcomes. The low proportion of poor functional outcomes (Supplementary Figure I) and the insignificant association with ALP in different stroke subtypes (Supplementary Table II) may account for the insignificant association between ALP levels and 3-month poor functional outcomes in our study. In addition, the percentages of the enrolled stroke patients with a history of stroke (27.5 vs. 34.0% by Zong et al. and 33.6% by Kim et al.), AF (6.6 vs. 23.7% by Kim et al. and 20.2% by Naito et al.) or pneumonia during hospitalization (6.7 vs. 8.3% by Zong et al.) were lower in the present study, and our patients tended to be younger (8, 18, 20). Previous stroke, AF, pneumonia, and old age are all potential factors leading to poor outcomes after stroke (24–26). These different outcomes must have caused our different findings from other studies, thus reaffirming the need for conducting more regional research.

The results of our subgroup analysis revealed that gender and drinking status did not affect the correlations between ALP levels and stroke outcomes, the findings being consistent with the CNSR-based study but not with some other studies (12, 18, 27, 28). By comparing clinical characteristics, we supposed that the imbalance in the female proportion (37.8 vs. 61.8%) and nondrinkers (76.3 vs. 96.1%) might contribute to the discrepancy, implying that gender and drinking status in our study should have some impact on the association between ALP and stroke outcomes (12). Therefore, further studies are needed to explore the effects of ALP on adverse stroke outcomes in subgroups of gender and alcohol consumption. In addition, the Kaplan–Meier curves of 3-month cumulative rates of all-cause mortality and recurrent stroke differ significantly in different stroke subtypes, providing a new direction for investigating ALP levels with all-cause mortality and recurrent stroke in different stroke types.

Although the mechanisms of the effect of ALP in stroke are currently not fully understood, some hypotheses have been proposed in the previous studies. One working hypothesis is that elevated ALP may accelerate the calcification and stiffening of vessels, decrease vascular compliance and cause atherosclerosis (9, 18, 29). Elevated ALP levels were also hypothesized to trigger systemic inflammation, subsequently leading to adverse stroke outcomes (30, 31). CD34-positive cells play an important role in maintaining vascular homeostasis and repair (32–34). A reduction in CD34-positive cells has been approved to be related to increased infarction numbers (35). Studies have reported that ALP level was positively related to the number of CD34-positive cells, therefore we speculated that low ALP level may lead to an increased likelihood of poor outcomes by affecting the number of CD34-positive cells (35, 36). Our results suggest that either higher or lower ALP levels are related to mortality and recurrence after stroke, providing new insight into the underlying mechanisms of the ALP impact on stroke outcomes.

This study has some limitations that should be addressed in future studies. First, the selection bias of enrolled patients existed as only patients with AS from the four tertiary grade A hospitals were included in this study. Such limited patient selection may limit the generalizability of our results for patients with AS in some other smaller community hospitals. Second, this study focused on the association of serum ALP levels within 24 h of admission and the 3-month outcome, so the potential influence of changes in ALP levels after discharge was not analyzed. Third, cerebrovascular and neuroimaging data were unavailable in this study, leading to a lack of image-associated risk factors. Fourth, the type of ALP being related to stroke outcomes could not be assessed because the data regarding ALP isozymes was incomplete in this study (16, 37). Further large-scale cohort studies in other populations and districts are needed to confirm the generalizability of our findings.

## Conclusion

In this multi-center, prospective cohort study, non-linear associations were observed between serum ALP levels and 3-month outcomes in patients with AS. Elevated serum ALP levels were associated with an increased risk of all-cause mortality and might be used as a qualified predictor of all-cause mortality in patients with AS within 3 months. The optimal range of ALP for reducing all-cause mortality was Q2 (63.0–76.7 U/L) with a nadir level of 70 U/L and, for reducing the risk of recurrent stroke, the optimal range of ALP was seen in Q3 (76.8–92.9 U/L). The results of our study suggest that the risk of 3-month all-cause mortality and recurrent stroke might be reduced by controlling ALP at preferable ranges in Xi'an district.

## Data Availability Statement

The raw data supporting the conclusions of this article will be made available by the authors, without undue reservation.

## Ethics Statement

The studies involving human participants were reviewed and approved by Ethics Committee of Xi'an No.1 Hospital. The patients/participants provided their written informed consent to participate in this study.

## Author Contributions

SW had full access to all of the data in the study and takes responsibility for the integrity of the data and the accuracy of the data analysis. WG and ZL planned and designed the study and wrote the manuscript. ZL contributed to the data cleaning and statistical analysis. QL, PL, YW, and QC contributed to follow-up patients and recorded the data at each stage. FW, XL, and JW revised the manuscript for important intellectual content. All authors have read and approved the final version of the manuscript.

## Funding

The study was supported by the Science and Technology Program of Shaanxi Province (No. 2021SF-333), the Science and Technology Plan Major Project of Xi'an city [No. 201805104YX12SF38(2)], the Science and Technology Plan Project of Xi'an city [No. 20YXYJ0008(1)] and the Scientific Research Project of Xi'an Health Commission (Nos. 2020ms03, 2020yb05, and 2021yb33). The funders had no role in the design and analysis of this trial.

## Conflict of Interest

The authors declare that the research was conducted in the absence of any commercial or financial relationships that could be construed as a potential conflict of interest.

## Publisher's Note

All claims expressed in this article are solely those of the authors and do not necessarily represent those of their affiliated organizations, or those of the publisher, the editors and the reviewers. Any product that may be evaluated in this article, or claim that may be made by its manufacturer, is not guaranteed or endorsed by the publisher.

## Supplementary Material

Supplementary Figure I Poor functional outcomes in different stroke subtypes at 3-month. AIS, acute ischemic stroke. TIA, transient ischemic attack; ICH, spontaneous intracerebral hemorrhage; SAH, subarachnoid hemorrhage.

Supplementary Figure II Stratified logistic regression analysis of serum ALP levels and 3-month outcomes. **(A)** All-cause mortality. **(B)** Recurrent stroke. **(C)** Poor functional outcome.

Supplementary Figure III Kaplan-Meier curves of 3-month outcomes among different stroke types. **(A)** All-cause mortality. **(B)** Recurrent stroke.
