# Supplementary figures and images for: Non-Linear Association Between Serum Alkaline Phosphatase and 3-Month Outcomes in Patients With Acute Stroke: Results From the Xi'an Stroke Registry Study of China
Source: Front Neurol. 2022 Jul 15;13:859258. doi: 10.3389/fneur.2022.859258 (PMC9334812; doi:10.3389/fneur.2022.859258)

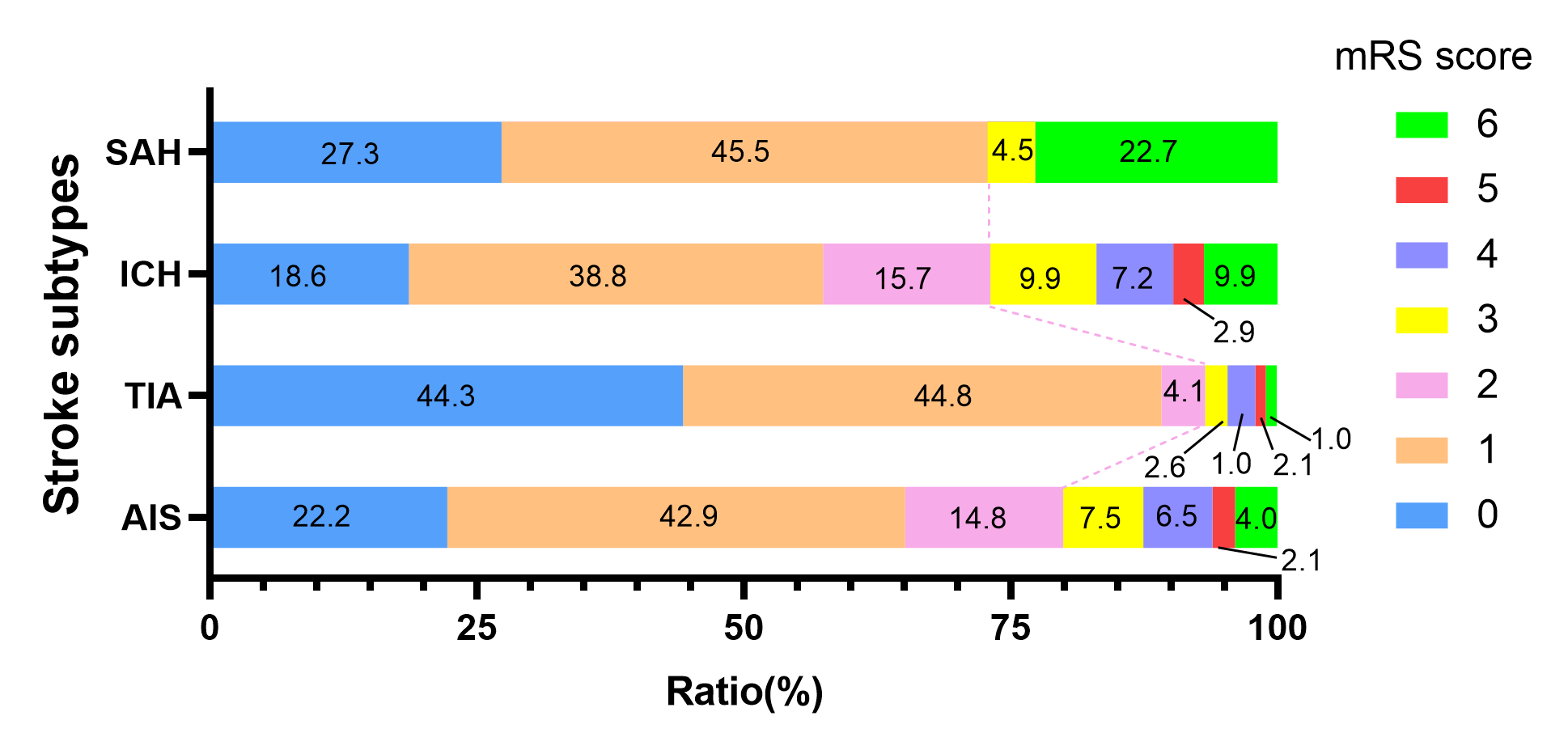

Supplement: Supplementary Figure I — Poor functional outcomes in different stroke subtypes at 3-month. AIS, acute ischemic stroke. TIA, transient ischemic attack; ICH, spontaneous intracerebral hemorrhage; SAH, subarachnoid hemorrhage. [file Image_1.TIF]

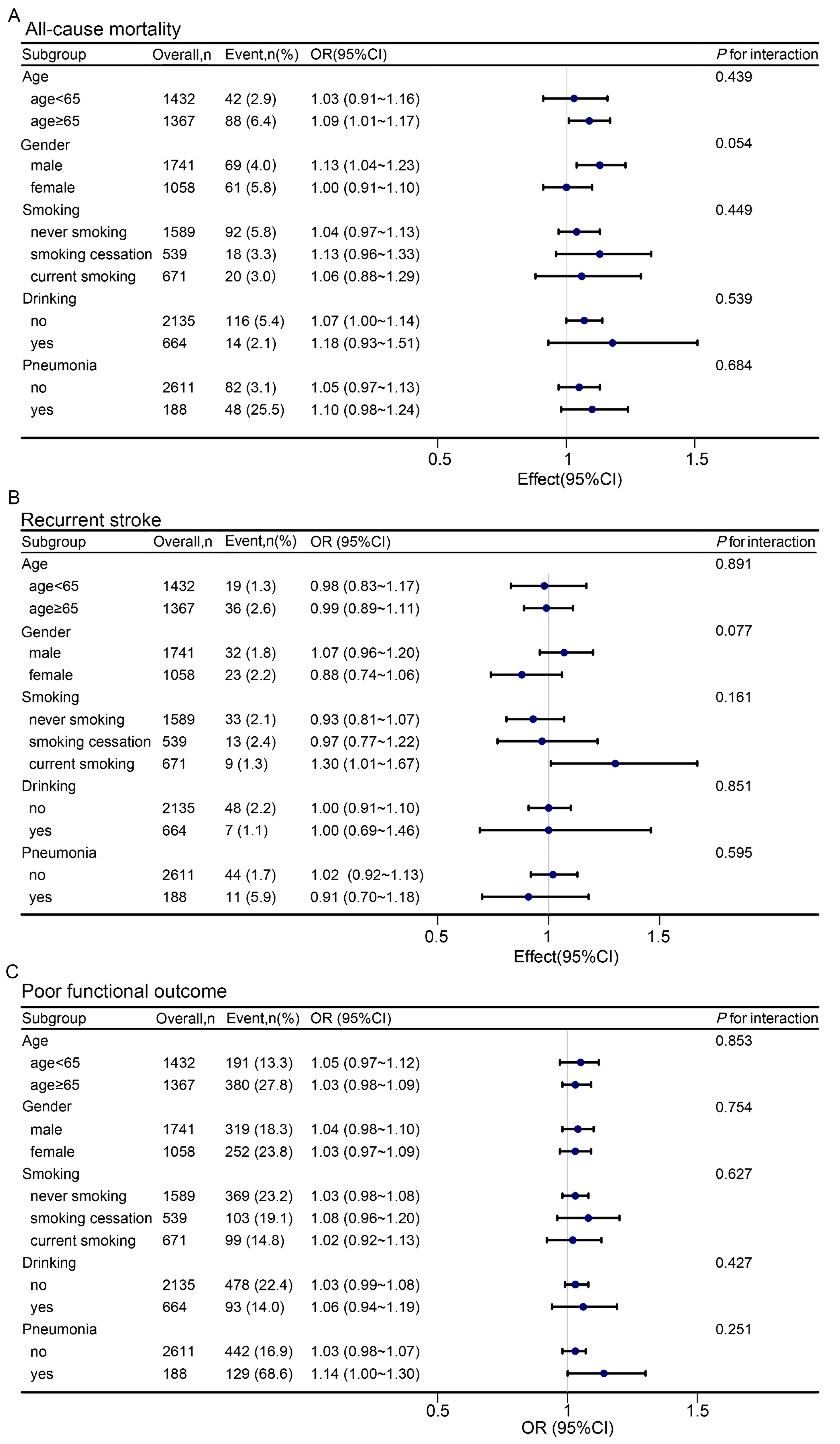

Supplement: Supplementary Figure II — Stratified logistic regression analysis of serum ALP levels and 3-month outcomes. (A) All-cause mortality. (B) Recurrent stroke. (C) Poor functional outcome. [file Image_2.TIF]

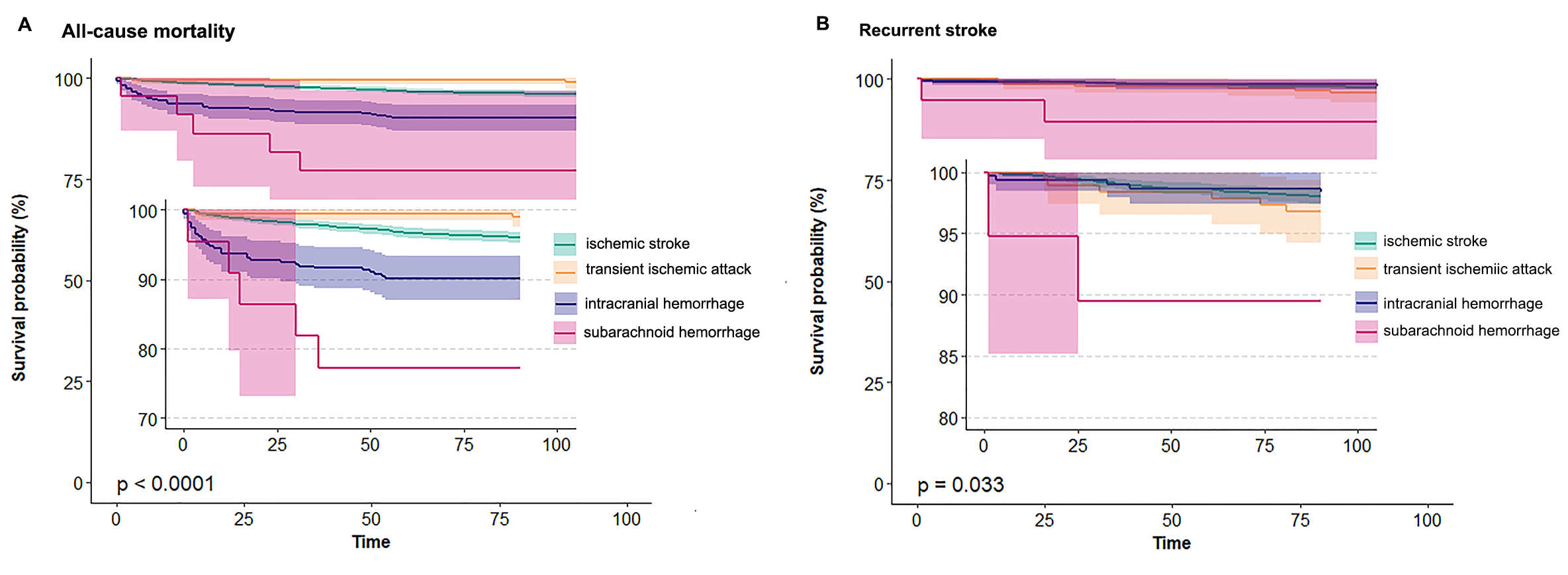

Supplement: Supplementary Figure III — Kaplan-Meier curves of 3-month outcomes among different stroke types. (A) All-cause mortality. (B) Recurrent stroke. [file Image_3.TIF]
