# Supplementary material for: Non-Linear Association Between Serum Alkaline Phosphatase and 3-Month Outcomes in Patients With Acute Stroke: Results From the Xi'an Stroke Registry Study of China
Source: Front Neurol. 2022 Jul 15;13:859258. doi: 10.3389/fneur.2022.859258 (PMC9334812; doi:10.3389/fneur.2022.859258)
Supplement: Supplementary file 4 [file Table_1.docx]

**Supplementary Table Ⅰ**| Threshold effect analysis of ALP level and three-month recurrent stroke

| Outcomes | OR (95%CI) | *P*-value |
| --- | --- | --- |
| One-line linear regression model (per 10-unit increase) | 1.00(0.91~1.10) | 0.978 |
| Two piecewise linear regression model |  |  |
| ALP < 80 U/L | 0.99 (0.97~1.02) | 0.675 |
| ALP >80 U/L | 1.02 (1.00~1.05) | 0.028 |
| Log-likelihood ratio test |  | 0.119 |

Notes: Adjusted for age, gender, smoking, drinking, previous stroke, National Institutes of Health Stroke Scale score, pneumonia during hospitalization, estimated glomerular filtration rate, white blood cell, body mass index, hypertension, diabetes mellitus and atrial fibrillation.

Abbreviations: ALP alkaline Phosphatase, OR odd ratio, CI confidence internal.

**Supplementary Table** **Ⅱ**| Logistic regression of the association between ALP and poor functional outcomes stratified by stroke subtypes

| **Stroke types** | **Overall, n** | **Event, n (%)** | **Crude OR (95%CI)** | ***P*-value** | **Adjusted^a^ OR (95% CI)** | | ***P*-value** | ***P* for interaction** |
| --- | --- | --- | --- | --- | --- | --- | --- | --- |
|  |  |  |  |  | |  |  | 0.573 |
| ischemic stroke | 2238 | 449 (20.1) | 1.01 (1.00~1.01) | 0.001 | | 1.00 (1.00~1.01) | 0.253 |  |
| transient ischemic attack | 194 | 13 (6.7) | 1.00 (0.98~1.02) | 0.856 | | 1.01 (0.98~1.04) | 0.436 |  |
| spontaneous intracerebral hemorrhage | 345 | 103 (29.9) | 1.01 (1.00~1.02) | 0.010 | | 1.01 (0.96~1.02) | 0.305 |  |
| subarachnoid hemorrhage | 22 | 6 (27.3) | 0.99 (0.96~1.03) | 0.747 | | 0.51 (0~Inf) | 0.999 |  |

^a^Adjusted for age, gender, smoking, drinking, previous stroke, National Institutes of Health Stroke Scale score, pneumonia during hospitalization, estimated glomerular filtration rate, white blood cell, body mass index, hypertension, diabetes mellitus and atrial fibrillation.

Abbreviations: OR odd ratio, CI confidence internal.
